# A retrospective study of risk factors, causative micro-organisms and healthcare resources consumption associated with prosthetic joint infections (PJI) using the Clinical Practice Research Datalink (CPRD) Aurum database

**DOI:** 10.1371/journal.pone.0282709

**Published:** 2023-03-21

**Authors:** Stefano Perni, Bsmah Bojan, Polina Prokopovich

**Affiliations:** School of Pharmacy and Pharmaceutical Sciences, Cardiff University, Cardiff, United Kingdom; Baqai Medical University, PAKISTAN

## Abstract

**Background:**

Prosthetic joint infection (PJI) is a serious complication after joint replacement surgery and it is associated with risk of mortality and morbidity along with high direct costs.

**Methods:**

The Clinical Practice Research Datalink (CPRD) data were utilized to quantify PJI incidence after hip or knee replacement up to 5 years after implant and a variety of risk factors related to patient characteristics, medical and treatment history along with characteristics of the original surgery were analyzed through Cox proportional hazard.

**Results:**

221,826 patients (individual joints 283,789) met all the inclusion and exclusion criteria of the study; during the study follow-up period (5 years), 707 and 695 PJIs were diagnosed in hip and knee, respectively. Patients undergoing joint replacement surgery during an unscheduled hospitalization had greater risk of PJI than patients whose surgery was elective; similarly, the risk of developing PJI after a secondary hip or knee replacement was about 4 times greater than after primary arthroplasty when adjusted for all other variables considered. A previous diagnosis of PJI, even in a different joint, increased the risk of a further PJI. Distribution of average LoS per each hospitalization caused by PJI exhibited a right skewed profile with median duration [IQR] duration of 16 days [8–32] and 13 days [7.25–32] for hip and knee, respectively. PJIs causative micro-organisms were dependent on the time between initial surgery and infection offset; early PJI were more likely to be multispecies than later (years after surgery); the identification of Gram- pathogens decreased with increasing post-surgery follow-up.

**Conclusions:**

This study offers a contemporary assessment of the budgetary and capacity (number and duration of hospitalizations along with the number of Accident and Emergency (A&E) visits) posed by PJIs in UK for the national healthcare system (NHS). The results to provide risk management and planning tools to health providers and policy makers in order to fully assess technologies aimed at controlling and preventing PJI. The findings add to the existing evidence-based knowledge surrounding the epidemiology and burden of PJI by quantifying patterns of PJI in patients with a relatively broad set of prevalent comorbidities.

## 1 Introduction

Total joint arthroplasty (TJA) has been reported to be one of the widely successful treatments for damaged hip or knee joints [1, 2]. However, TJA failure are observed as consequence of periprosthetic joint infections (PJIs) [3, 4]. The reported rate of PJI is one to two percent after primary TJA despite prevention and management policies [5, 6]. PJI prevention mainly relys on antimicrobial prophylactic therapy either systemically or in situ from antibiotic laden bone cement; Management can involve debridement, removal of the implant and his replacement or, in extreme cases, amputation; furthermore, PJI can also lead to death [7]. Therefore, this devastating complication is associated with repeated hospital admissions, severe pain, long term functional deficit and poor health outcomes along with a significant economic burden [8–10].

Hence, it becomes increasingly important to understand the risk factors, both modifiable and non-modifiable, for PJI incidence and outcomes to optimize medical management in patients, scheduled to undergo TJA, at high risk of periprosthetic infection [11, 12]. Numerous risk factors have been identified for PJI after TJA including obesity [13, 14], diabetes mellitus [15, 16], rheumatoid arthritis (RA) [17], urinary tract infections [18], operation time [14]. However, not all studies have demonstrated a similar association between these variables and PJI [19] and, generalization of the conclusions obtained from patients of a certain country may not be applicable to another [20–24].

Contemporary UK specific incidence rates, specific treatment pathways and healthcare related resources consumption have not been well established yet. This information is critical in the assessment of new approaches to prevention and management of PJI thus improving both patient satisfaction post procedure and return on the limited resources of the national healthcare system (NHS).

In this study, our purpose was to identify nationally representative, and current, risk factors for developing PJI after total hip or knee arthroplasty from a large pool of potential covariates that covers type and characteristics of the joint replacement surgery along with patients’ demographic, medical and treatment history. Clinical Practice Research Datalink (CPRD) [25–27] with linkage with Hospital episode statistics (HES) offers the opportunity to explore a wider range of the risk factors for PJI than those recorded in only secondary care [28–30]. Moreover, we employed these data to analyze the reported causative micro-organisms of PJI and to determine the present-day burden posed by PJIs on the NHS in terms of overall cost, length of stay and number of hospital admissions and Accident and Emergency (A&E) visits.

## 2 Methods

### 2.1 Data sources

Data were obtained from the UK Clinical Practice Research Datalink (CPRD) with linkage to Inpatient and Outpatient Hospital Episode Statistics (HES) secondary care data and Office for National Statistics (ONS) mortality data.

### 2.2 Study design

This was a retrospective study of patients undergoing hip or knee replacement; the index date for inclusion was the day of joint replacement surgery. Patients were included if relevant inclusion were met: Aged at least 30 years at index date and surgery after 01/01/2007.

### 2.3 Sample size considerations

This research was largely descriptive rather than inferential in nature in that it aimed to characterise pattens of PJI. A sample size and power calculation were, therefore, not undertaken with respect to identifying differences in outcomes between groups. However, the number of patients identified suggests that the analysed population will be large enough to allow for sufficiently robust conclusions to be drawn from the study.

### 2.4 Participants

Patients undergoing hip or knee replacement surgery were identified through OPCS codes for such procedures in the HES database (S1 Table).

Patients were excluded if they meet any of the following criteria: aged <30 years at index date, registered on the CPRD for < 6 months prior to index date and most recent CPRD up-to-standard (UTS) date > 6 months prior to index date. Laterality of the procedure was determined through OPCS code (Z94.2: Right sided operation and Z94.3: Left sided operation); when the code reported a bilateral procedure (Z94.1: Bilateral operation) two separate entries were created, one for the left joint and one for the left. A specific entry was created for each patient, index date, joint (hip or knee) and side (left or right).

Eligible patients were followed-up from index date and their records extracted for all observations up to and including the first occurrence of: joint replaced, death, loss to follow-up or end of study period (5-years after last joint replacement surgery).

Arthroplasty were defined as primary if this specifically stated in the OPCS procedure code, the procedures were categorized as secondary when specifically stated in the OPCS procedure code or if an arthroplasty procedure (S1 Table) was observed in the patient record in the same joint; otherwise the procedure primary or secondary property was classified as “unknown”.

The date of replacement was determined as the date a subsequent arthroplasty procedure was recorded on the same joint. This also corresponded to the index date of a further entry.

Loss to follow-up was be defined as the earliest date a patient was transferred out of the practice or the date that the practice left the CPRD database.

Covariate related to patient characteristics were extracted from the CPRD database; joint replacement surgery properties were derived from the HES database according to OPCS codes (for type of fixation, laterality fixation, grafts…) and admission codes. Medical history was determined by the presence/absence of disease specific MedCodeID codes in the CPRD database or of ICD-10 codes in the HES database before index date.

Prescription history was assessed from relevant BNF codes reported in the CPRD database.

### 2.5 Primary and secondary outcomes

The primary outcome of the study was the determination of risk factors for PJI: PJI occurrence determined through the presence of the ICD-10 code M84.5 in the HES database. In order to ascertain that PJI diagnosed were affecting the joint of interest as patients could have multiple joints replaced, only a diagnosis of PJI and a record any procedure (OPCS) in the joint of interest during the hospitalization were considered (S2 Table).

The date of PJI offset was determined as the hospitalization date corresponding to the first diagnosis of PJI in the joint (hip or knee and left or right). The time to PJI was calculated as difference between index date and date of PJI occurrence.

Secondary outcomes of the study were the quantification of:

- Number of hospitalizations and A&E visits, identified from the HES records as unique hospitalization identifier codes and the admission origin code;
- Length of Stay (LoS) of each hospitalization, calculated as the number of days between admission and discharge.
- Procedures performed during hospitalization caused by PJI, determined using OPCS codes;
- Direct costs of procedures associated to PJI, determined using the NHS reference cost [31].
- Final outcome of each PJI, assessed based on procedures performed (S2 Table) and categorized as DAIR if no device replacement was carried out on the joint of interests during any hospitalization with a diagnosis of PJI.
- Causative micro-organisms of PJI, determined through the ICD-10 records in HES database.

### 2.6 Comparison group(s) or controls

No comparison or control groups are specified. Patients will be characterised by levels of demographic, clinical and treatment characteristics.

### 2.7 Ethics

This study protocol (19_009) was reviewed by the Independent Scientific Advisory Committee (ISAC) and received approval in January 2020; a minor amendment was approved in December 2021. The research team had access only to data de-identified before delivery thus approval from an institutional review board was not sought.

### 2.8 Statistical analysis

Descriptive analyses were generated, characterizing patient demographics, clinical and treatment characteristics. Summary statistics (for example, mean, standard deviation, standard error, median, inter-quartile range, minimum, and maximum) were calculated for continuous variables, and number and proportion/percentage for categorical variables. The number and proportion of patients with missing data was also reported for each of the variables of interest.

Where to statistically describe differences between patient subgroups was appropriate, univariate methods were employed. The type of test used was dependent on the type/distribution of the outcome variable. t-test was used for numeric variables and chi-squared tests was be used for (unordered) categorical variables. A p-value < 0.05 was considered statistically significant.

Risk factors for PJI were studies using the Cox proportional hazard model both in its univariate and multivariate form.

LoS and number of hospitalizations were fitted with negative binomial and Poisson distributions, along the zero inflated and zero truncated form, respectively. The final model selection was based on the observation of goodness of fitting parameters such as AIC, BIC and Log-likelihood.

All data collection, analysis and visualization were performed using R (ver 4.0) and relevant packages [32, 33].

## 3 Results

In total 235,249 patients, corresponding to 330,173 joints, were detected in the CPRD database after linkage to HES and ONS who underwent hip or knee replacement with non-missing laterality of the surgery. 221,826 patients (individual joints 283,789) met all the inclusion and exclusion criteria of the study (S1 Table).

Most of the patients completed the observational period of 5 years; the most common reason for patients (n = 76,800) to be lost to follow-up was the last collection date in the database being earlier than 5 years from surgery. The second and third most common reason for not completing the 5 years follow-up period were patients transferring out of CPRD (n = 35,831) and death (n = 32,981), respectively (S4 Table).

### 3.1 Baseline characteristics

Patients in the cohort were predominantly female, with age ranging from 31 to 109 years and a median of 72 years. Majority of patients had a BMI between 26 and 30 with a median BMI record of 28.74 at the time of surgery to replace the hip or knee joint; for about 30% of patients no record of BMI was available (Table 1). Gender distribution was different between the patients who developed PJI and those who did not (p < 0.001), majority of patients diagnosed with PJI in the joint replaced were male. Age and BMI were also parameters with different distribution in the two cohorts regardless of considering these variable continuous or categorical. Patient developing PJI were generally younger and with higher BMI (p < 0.001).

**Table 1:** Characteristics of patients at the time of hip or knee implant surgery.

Variable	All	No PJI	PJI	p value
Gender				
Female	173,961 (61.30%)	173,291 (61.37%)	670 (47.79%)	<0.001
Male	109,828 (38.70%)	109,096 (38.63%)	732 (52.21%)	
Age				
≤ 45	4,074 (1.44%)	4,027 (1.43%)	47 (3.35%)	<0.001
46–55	17,512 (6.17%)	17,392 (6.16%)	120 (8.56%)	
56–65	54,632 (19.25%)	54,279 (19.22%)	353 (25.18%)	
66–75	96,747 (34.09%)	96,262 (34.09%)	485 (34.59%)	
76–85	81,892 (28.86%)	81,540 (28.88%)	352 (25.11%)	
> 85	28,932 (10.19%)	28,887 (10.23%)	45 (3.21%)	
Mean (SD)	71.88	71.90 (10.98)	68.40 (10.86)	<0.001
Median	72	72	69	
IQR	65–80	65–80	62–76	
Min, max	31, 109	31, 109	31, 98	
BMI				
< 20	7,694 (2.71%)	7,675 (2.72%)	19 (1.36%)	<0.001
20–25	39,004 (13.74%)	38,873 (13.77%)	131 (9.34%)	
26–30	70,293 (24.77%)	69,987 (24.78%)	306 (21.83%)	
31–35	50,075 (17.65%)	49,776 (17.63%)	299 (21.33%)	
36–50	30,963 (10.91%)	30,736 (10.88%)	227 (16.19%)	
> 50	698 (0.25%)	691 (0.24%)	7 (0.50%)	
Unknown	85,062 (29.97%)	84,649 (29.98%)	413 (29.46%)	
Mean (SD)	29.37	29.36 (6.02)	31.20 (6.28)	<0.001
Median	28.74	28.73	30.6	
IQR	25.30–32.86	25.30–32.81	27.00–34.80	
Min, max	10.46, 99.90	10.46, 99.90	13.10, 60.80	
Smoking status				
Non-smoker	153,344 (54.03%)	152,682 (54.07%)	662 (47.22%)	<0.001
Cigar	223 (0.08%)	> 218	< 5	
Current	12,098 (4.26%)	12,028 (4.26%)	70 (4.99%)	
Ex-smoker	88,563 (31.21%)	88,051 (31.18%)	512 (36.52%)	
Heavy	307 (0.11%)	> 302	< 5	
Light	782 (0.28%)	> 778	< 5	
Moderate	704 (0.25%)	> 699	< 5	
Quitting	886 (0.31%)	881 (0.31%)	5 (0.36%)	
Unknown	26,882 (9.47%)	26,738 (9.47%)	144 (10.27%)	
Alcohol consumption				
Non-drinker	4,826 (1.70%)	4,810 (1.70%)	16 (1.14%)	0.09
Ex drinker	418 (0.15%)	> 413	< 5	
Heavy drinker	1,580 (0.56%)	1,569 (0.56%)	11 (0.78%)	
Light drinker	52,731 (18.58%)	52,446 (18.57%)	285 (20.33%)	
Moderate drinker	9,282 (3.27%)	9,223 (3.27%)	59 (4.21%)	
Social drinker	4,781 (1.68%)	4,752 (1.68%)	29 (2.07%)	
Very heavy	561 (0.20%)	> 556	< 5	
Other	1,732 (0.61%)	1,723 (0.61%)	9 (0.64%)	
Unknown	207,878 (73.25%)	206,892 (73.27%)	986 (70.33%)	

More than half of patients were non-smokers and about 30% had quit the habit at the time of joint replacement surgery; less the 10% had no record regarding their smoking habit. The smoking status was different among patients developing PJI compared to patients not experiencing PJI in the 5 years post implant surgery (p < 0.001). Patients attitude toward alcohol consumption did not appear to impact the risk of PJI (p> 0.05) (Table 1).

Similar number of patients underwent total hip or knee replacement, replacement of only the femur head was the least common procedure among those considered and the resulted in the lowest risk of developing PJI (p < 0.001). The implanted device was fixed with bone cement in 65.3% of patients while 23.4% of devices implanted were uncemented; hybrid fixation was used in 7.9% of procedures; the fixation method distribution among patients who developed PJI was different than those who did not (p < 0.001). Over 90% of recorded procedures were primary arthroplasty, however PJI were more likely to develop after replacement procedures (p < 0.001). 82.8% of procedures were elective and bone grafts were recorded in under 2% of surgeries and in knee replacement patellar resurfacing was observed in 14.9% of joints, both surgical characteristics distribution among the cohorts of patients with PJI and without-PJI were statistically different (p < 0.001) (Table 2).

**Table 2:** Characteristics of implant surgery.

Variable	All	No PJI	PJI	p value
Joint replaced				
Femur	38,159 (13.45%)	38,102 (13.49%)	57 (4.07%)	<0.001
Hip	124,265 (43.79%)	123,615 (43.78%)	650 (46.36%)	
Knee	121,365 (42.77%)	120,670 (42.73%)	695 (49.57%)	
Laterality of operation				
Left	133,269 (46.96%)	132,578 (46.95%)	691 (49.29%)	0.08
Right	150,520 (53.04%)	149,809 (53.05%)	711 (50.71%)	
Type of fixation				
Cemented	185,253 (65.28%)	184,327 (65.27%)	926 (66.05%)	<0.001
Hybrid	22,349 (7.88%)	22,261 (7.88%)	88 (6.28%)	
Non cemented	66,507 (23.44%)	66,202 (23.44%)	305 (21.75%)	
Unknown	9,680 (3.41%)	9,597 (3.40%)	83 (5.92%)	
Primary arthroplasty				
Yes	262,247 (92.41%)	261,304 (92.53%)	943 (67.26%)	<0.001
No	20,817 (7.34%)	20,371 (7.21%)	446 (31.81%)	
Unknown	725 (0.26%)	712 (0.25%)	13 (0.93%)	
Admission type				
Elective	234,961 (82.79%)	233,778 (82.79%)	1,183 (84.38%)	0.03
A&E	48,156 (16.97%)	47,944 (16.98%)	212 (15.12%)	
Unknown	672 (0.24%)	665 (0.24%)	7 (0.50%)	
Patella resurfacing (* only for knee replacement surgeries)				
No	103,244 (85.07%)	102,605 (85.03%)	639 (91.94%)	<0.001
Yes	18,121 (14.93%)	18,065 (14.97%)	56 (8.06%)	
Graft				
No	279,681 (98.55%)	278,322 (98.56%)	1,359 (96.93%)	<0.001
Autograft	1,857 (0.65%)	1,841 (0.65%)	16 (1.14%)	
Autograft + other	76 (0.03%)	> 71	< 5	
Other graft	2,175 (0.77%)	2,149 (0.76%)	26 (1.85%)	

The most common comorbidity observed in the patients cohort was osteoarthritis (OA) (n = 199,551–70.32%) followed by hypertension (n = 157,086–55.35%) and cancer (n = 135,577–47.77%); other diseases affecting more than 10% of the included patients at the time of arthroplasty were: rheumatoid arthritis (n = 72,090, 25.40%), CKD (n = 45,916–16.18%), Type1 or Type2 diabetes (n = 42,259–14.89%), anemia (40,062–14.12%) and ischemic heart disease (n = 31,192–10.99%). Of these, CKD, diabetes, OA and RA frequency on patients affected by PJI was statistically different (p < 0.01) than those who did not develop PJI. Patients affected by DVT or dementia at the time of joint replacement surgery were more likely to have developed PJI (p < .001); while osteoporosis at the time of surgery was more common in patients that did not develop PJI (p < 0.001). The majority of patients had been diagnosed with 2 or 3 of the comorbidities considered in this work at the time of surgery, more than a quarter of the cohort had been diagnosed with 4 or 5 of the diseases considered. The overall number of diagnosed comorbidities did not differ between the patients group developing PJI compared to those who did not (p > 0.05). A previous diagnosis of PJI before surgery was more common in the patients that developed a further PJI even if the infection was in a different joint (p < 0.001) (Table 3).

**Table 3:** Medical history of patients at the time of hip or knee implant surgery.

Variable	All	No PJI	PJI	p value
AF				
No	258,233 (90.99%)	256,981 (91.00%)	1,252 (89.30%)	0.03
Yes	25,556 (9.01%)	25,406 (9.00%)	150 (10.70%)	
Liver failure				
No	283,035 (99.73%)	281,638 (99.73%)	1,397 (99.64%)	0.69
Yes	754 (0.27%)	749 (0.27%)	5 (0.36%)	
CKD				
No	237,873 (83.82%)	236,657 (83.81%)	1,216 (86.73%)	<0.01
Yes	45,916 (16.18%)	45,730 (16.19%)	186 (13.27%)	
PE				
No	277,125 (97.65%)	275,767 (97.66%)	1,358 (96.86%)	0.06
Yes	6,664 (2.35%)	6,620 (2.34%)	44 (3.14%)	
DVT				
No	272,228 (95.93%)	270,911 (95.94%)	1,317 (93.94%)	<0.001
Yes	11,561 (4.07%)	11,476 (4.06%)	85 (6.06%)	
Diabetes				
No	241,530 (85.11%)	240,385 (85.13%)	1,145 (81.67%)	<0.001
Yes	42,259 (14.89%)	42,002 (14.87%)	257 (18.33%)	
OA				
No	84,238 (29.68%)	83,868 (29.70%)	370 (26.39%)	<0.01
Yes	199,551 (70.32%)	198,519 (70.30%)	1,032 (73.61%)	
RA				
No	211,699 (74.60%)	210,702 (74.61%)	997 (71.11%)	<0.01
Yes	72,090 (25.40%)	71,685 (25.39%)	405 (28.89%)	
AC				
No	148,212 (52.23%)	147,443 (52.21%)	769 (54.85%)	0.05
Yes	135,577 (47.77%)	134,944 (47.79%)	633 (45.15%)	
HF				
No	276,416 (97.40%)	275,045 (97.40%)	1,371 (97.79%)	0.41
Yes	7,373 (2.60%)	7,342 (2.60%)	31 (2.21%)	
MI				
No	256,242 (90.29%)	255,002 (90.30%)	1,240 (88.45%)	0.02
Yes	27,547 (9.71%)	27,385 (9.70%)	162 (11.55%)	
IHD				
No	252,597 (89.01%)	251,370 (89.02%)	1,227 (87.52%)	0.08
Yes	31,192 (10.99%)	31,017 (10.98%)	175 (12.48%)	
Hypertension				
No	126,703 (44.65%)	126,102 (44.66%)	601 (42.87%)	0.19
Yes	157,086 (55.35%)	156,285 (55.34%)	801 (57.13%)	
COPD				
No	262,628 (92.54%)	261,329 (92.54%)	1,299 (92.65%)	0.92
Yes	21,161 (7.46%)	21,058 (7.46%)	103 (7.35%)	
Hemorrhagic stroke				
No	277,864 (97.91%)	276,488 (97.91%)	1,376 (98.15%)	0.6
Yes	5,925 (2.09%)	5,899 (2.09%)	26 (1.85%)	
Ischemic stroke				
No	276,570 (97.46%)	275,198 (97.45%)	1,372 (97.86%)	0.38
Yes	7,219 (2.54%)	7,189 (2.55%)	30 (2.14%)	
Dementia				
No	273,956 (96.54%)	272,571 (96.52%)	1,385 (98.79%)	<0.001
Yes	9,833 (3.46%)	9,816 (3.48%)	17 (1.21%)	
Thyroidism				
No	281,401 (99.16%)	280,008 (99.16%)	1,393 (99.36%)	0.5
Yes	2,388 (0.84%)	2,379 (0.84%)	9 (0.64%)	
Anemia				
No	243,727 (85.88%)	242,523 (85.88%)	1,204 (85.88%)	1
Yes	40,062 (14.12%)	39,864 (14.12%)	198 (14.12%)	
Osteoporosis				
No	259,856 (91.57%)	258,531 (91.55%)	1,325 (94.51%)	<0.001
Yes	23,933 (8.43%)	23,856 (8.45%)	77 (5.49%)	
PJI before in same joint				
No	282,212 (99.44%)	280,936 (99.49%)	1,276 (91.01%)	<0.001
Yes	1,577 (0.56%)	1,451 (0.51%)	126 (8.99%)	
PJI before in another joint (* not only hip or knee)				
No	280,664 (98.90%)	279,402 (98.94%)	1,262 (90.01%)	<0.001
Yes	3,125 (1.10%)	2,985 (1.06%)	140 (9.99%)	
Total number of comorbidities				
0–1	53,909 (19.00%)	53,652 (19.00%)	257 (18.33%)	0.15
2–3	127,623 (44.97%)	127,018 (44.98%)	605 (43.15%)	
4–5	74,689 (26.32%)	74,303 (26.31%)	386 (27.53%)	
6–7	22,085 (7.78%)	21,958 (7.78%)	127 (9.06%)	
8 or more	5,483 (1.63%)	5,456 (1.63%)	27 (1.85%)	

The only medications used by more than half of patients at any time before the surgery were antibacterial and NSAID; antifungal drugs were used by about 20% of patients before arthroplasty and steroids were prescribed to about 10% of the full cohort; the most common anticoagulant treatment observed was warfarin. Majority of patients receiving an intra-articular injection in the joint of interest did receive only 1 injection and more likely more than 6 months before surgery; some patients had received over 5 intra-articular injections in the joint of interest. Patients in the cohort had also received intra-articular injections in other joints, if they received these injections, the most common number of procedures undergone was 3 and the last recorded more than 6 months before the index surgery (Table 4).

**Table 4:** Treatment history of patients at the time of hip or knee implant surgery.

Variable	All	No PJI	PJI	p value
Steroids injection				
No use	259,053 (91.28%)	257,798 (91.29%)	1,255 (89.51%)	0.11
Use < 3 months	1,368 (0.48%)	1,359 (0.48%)	9 (0.64%)	
Use 3–6 months	2,568 (0.90%)	2,551 (0.90%)	17 (1.21%)	
Use > 6 months	20,800 (7.33%)	20,679 (7.32%)	121 (8.63%)	
Chondroitin glucosamine				
No use	280,998 (99.02%)	279,613 (99.02%)	1,385 (98.79%)	0.73
Use < 3 months	493 (0.17%)	> 488	< 5	
Use 3–6 months	135 (0.05%)	> 130	< 5	
Use > 6 months	2,163 (0.76%)	2,149 (0.76%)	14 (1.00%)	
NSAID				
No use	124,717 (43.95%)	124,184 (43.98%)	533 (38.02%)	<0.001
Use < 3 months	55,388 (19.52%)	54,966 (19.46%)	422 (30.10%)	
Use 3–6 months	15,509 (5.46%)	15,421 (5.46%)	88 (6.28%)	
Use > 6 months	88,175 (31.07%)	87,816 (31.10%)	359 (25.61%)	
Methotrexate				
No use	277,084 (97.64%)	275,730 (97.64%)	1,354 (96.58%)	0.04
Use < 3 months	4,304 (1.52%)	4,270 (1.51%)	34 (2.43%)	
Use 3–6 months	236 (0.08%)	> 231	< 5	
Use > 6 months	2,165 (0.76%)	2,152 (0.76%)	13 (0.93%)	
DMARD				
No use	279,519 (98.50%)	278,140 (98.50%)	1,379 (98.36%)	0.03
Use < 3 months	2,311 (0.81%)	2,305 (0.82%)	6 (0.43%)	
Use 3–6 months	231 (0.08%)	< 226	< 5	
Use > 6 months	1,728 (0.61%)	1,714 (0.61%)	14 (1.00%)	
Antibacterial				
No use	83,025 (29.26%)	82,630 (29.26%)	395 (28.17%)	0.03
Use < 3 months	54,748 (19.29%)	54,454 (19.28%)	294 (20.97%)	
Use 3–6 months	24,826 (8.75%)	24,680 (8.74%)	146 (10.41%)	
Use > 6 months	121,190 (42.70%)	120,623 (42.72%)	567 (40.44%)	
Antifungal				
No use	224,414 (79.08%)	223,345 (79.09%)	1,069 (76.25%)	0.02
Use < 3 months	7,530 (2.65%)	7,478 (2.65%)	52 (3.71%)	
Use 3–6 months	4,388 (1.55%)	4,363 (1.55%)	25 (1.78%)	
Use > 6 months	47,457 (16.72%)	47,201 (16.72%)	256 (18.26%)	
DOAC				
No use	278,363 (98.09%)	277,001 (98.09%)	1,362 (97.15%)	0.01
Use < 3 months	3,725 (1.31%)	3,694 (1.31%)	31 (2.21%)	
Use 3–6 months	303 (0.11%)	> 298	< 5	
Use > 6 months	1,398 (0.49%)	1,389 (0.49%)	9 (0.64%)	
Heparin				
No use	279,872 (98.62%)	278,494 (98.62%)	1,378 (98.29%)	0.06
Use < 3 months	587 (0.21%)	582 (0.21%)	5 (0.36%)	
Use 3–6 months	261 (0.09%)	> 256	< 5	
Use > 6 months	3,069 (1.08%)	3,054 (1.08%)	15 (1.07%)	
Warfarin				
No use	265,204 (93.45%)	263,922 (93.46%)	1,282 (91.44%)	0.01
Use < 3 months	12,094 (4.26%)	12,021 (4.26%)	73 (5.21%)	
Use 3–6 months	1,081 (0.38%)	1,073 (0.38%)	8 (0.57%)	
Use > 6 months	5,410 (1.91%)	5,371 (1.90%)	39 (2.78%)	
Intra-articular injections (n)				
0	271,827 (95.78%)	270,497 (95.79%)	1,330 (94.86%)	0.16
1	8,389 (2.96%)	8,337 (2.95%)	52 (3.71%)	
2	2,197 (0.77%)	2,188 (0.77%)	9 (0.64%)	
3	721 (0.25%)	716 (0.25%)	5 (0.36%)	
4	264 (0.09%)	> 259	< 5	
5	178 (0.06%)	> 173	< 5	
> 5	213 (0.08%)	> 208	< 5	
Last intra-articular injection				
No use	271,827 (95.78%)	270,497 (95.79%)	1,330 (94.86%)	0.37
Use < 3 months	434 (0.15%)	> 429	< 5	
Use 3–6 months	1,805 (0.64%)	1,794 (0.64%)	11 (0.78%)	
Use > 6 months	9,723 (3.43%)	9,664 (3.42%)	59 (4.21%)	
Intra-articular injection in other joints (n)				
0	249,945 (88.07%)	248,747 (88.09%)	1,198 (85.45%)	<0.01
1	294 (0.10%)	> 289	< 5	
2	3,901 (1.37%)	3,873 (1.37%)	28 (2.00%)	
3	9,606 (3.38%)	9,564 (3.39%)	42 (3.00%)	
4	6,541 (2.30%)	6,497 (2.30%)	44 (3.14%)	
5	2,090 (0.74%)	2,078 (0.74%)	12 (0.86%)	
> 5	11,412 (4.02%)	11,335 (4.01%)	77 (5.49%)	
Last intra-articular injection in other joints				
No use	249,945 (88.07%)	248,747 (88.09%)	1,198 (85.45%)	<0.01
Use < 3 months	1,266 (0.45%)	1,256 (0.44%)	10 (0.71%)	
Use 3–6 months	4,031 (1.42%)	4,012 (1.42%)	19 (1.36%)	
Use > 6 months	28,547 (10.06%)	28,372 (10.05%)	175 (12.48%)	

### 3.2 Primary outcome—PJI risk factors

**Fig 1:**
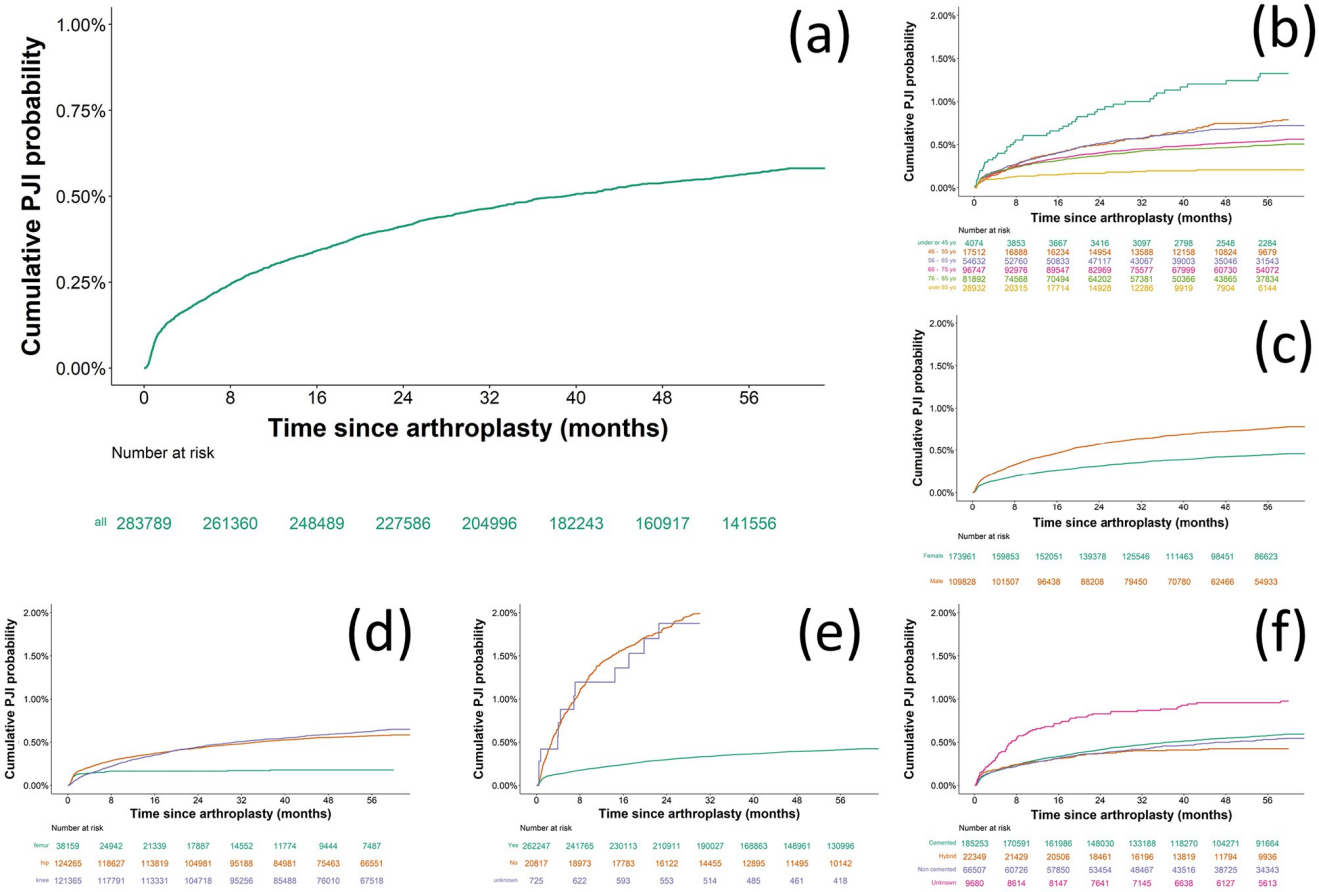
Example of Kaplan-Meier curves of cumulative risk of PJI for the entire cohort (a) and stratified based on age (b), gender (c), joint (d), primary/replacement surgery (e) and type of fixation (f).

During the study follow-up period, 707 and 695 PJIs were diagnosed in hip and knee respectively. The cumulative incidence of PJI is reported in the Kaplan-Meier curve in Fig 1a; 0.21% of the considered joints developed PJI in the first 6 months after surgery, 0.30% in the first year, 0.41% in the first 2 years, 0.54% in the first 4 years and 0.58% in the first 5 years. Therefore, about of a third of the recorded PJI were diagnosed in the first 6 months and half in the first year. Examples of Kaplan- Meier estimates are presented in Fig 1; older patients hazard of developing PJI was lower than younger ones (log-rank p < 0.001); similarly, male patients developed PJI at a higher rate than female (log-rank p < 0.001) (Fig 1b and 1c). The rate of PJI development in total hip and knee joints was not statistically different (log-rank p > 0.05), but greater than when only femur head was replaced (log-rank p < 0.001) (Fig 1d). Joint after primary replacement had a lower rate of PJI development than after revision (log-rank p < 0.001) or surgeries where the primary or replacement characteristic could not be ascertained (Fig 1e). In joints with an unknown fixation method the rate of PJI development was greater than any of method of fixation (log-rank p < 0.001) (Fig 1f).

**Fig 2:**
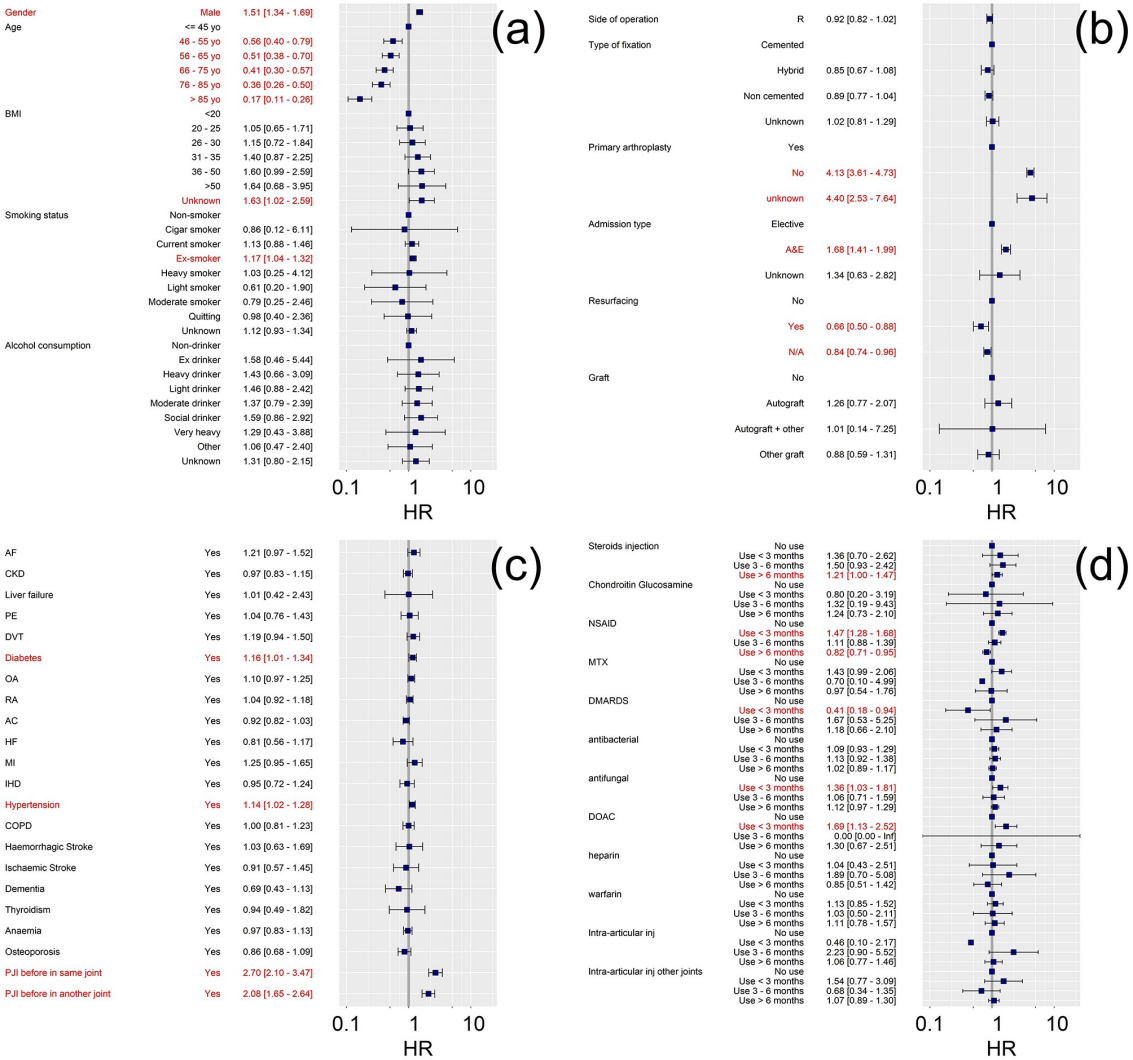
Forest plot of Hazard ratios (HR) of multivariate Cox proportional regression model for variables related to patient characteristics (a), arthroplasty surgery (b), medical history (c) and drug history (d).

When patients’ risk of developing PJI was fitted with the Cox regression proportional hazard model, male patients had a greater risk of developing PJI than female, also the older the patient at the time of surgery the lower the risk of been diagnosed with a PJI after the procedure. Other patients baseline characteristics such as BMI were not a risk factor for PJI when adjusted for other variables (Fig 2 and S1 Fig).

Patients undergoing joint replacement surgery during a hospitalization after been admitted to A&E had greater risk of PJI than patients whose surgery was elective; similarly, the risk of developing PJI after a secondary hip or knee replacement was about 4 times greater than after primary arthroplasty when adjusted for all other variables considered. Patellar resurfacing was associated to a reduced the risk of PJI after knee replacement while the type of fixation method did not have a statistically significant impact on the adjusted hazard rate estimated by the Cox regression model (Fig 2 and S1 Fig).

Numerous comorbidities, such as AF, RA, PE and DVT, were risk factors for PJI following hip or knee replacement when the considered individually in the Cox regression (S1 Fig); however only diabetes and hypertension remained statistically significant in the fully adjusted model (Fig 2). A previous diagnosis of PJI, even in a different joint, increased the risk of a further PJI.

Prescription of DOACs or antifungal in the 3 months before joint replacement surgery increased the risk of patients developing PJI while a prescription of DMARDs in the same period reduced the risk of PJI. Intra-articular injections before implant surgery or prescription of other anticoagulant drugs, such as warfarin and heparin, did not have a statistically significant impact on the adjusted hazard ratio estimated by the Cox model (Fig 2 and S1 Fig).

### 3.3 Secondary outcomes

#### 3.3.1 PJI health resource consumption and outcomes

Distribution of average LoS per each hospitalization caused by PJI exhibited a right skewed profile with mean values of 26.5 and 18.4 days for hip and knee respectively; median [IQR] duration were 16 [8–32] and 13 [7.25–32] for hip and knee respectively.

The average length of stay (LoS) of each hospitalization following a PJI diagnosis after index surgery was modelled with Poisson and negative binomial distributions and their relative zero inflated version. The fitting of the data with the zero inflated version of the distributions was better than the plain formulation of the distribution as described by greater Log-likelihood and lower AIC and BIC coefficients (S5 Table); negative binomial zero inflated gave the overall better performance.

**Fig 3:**
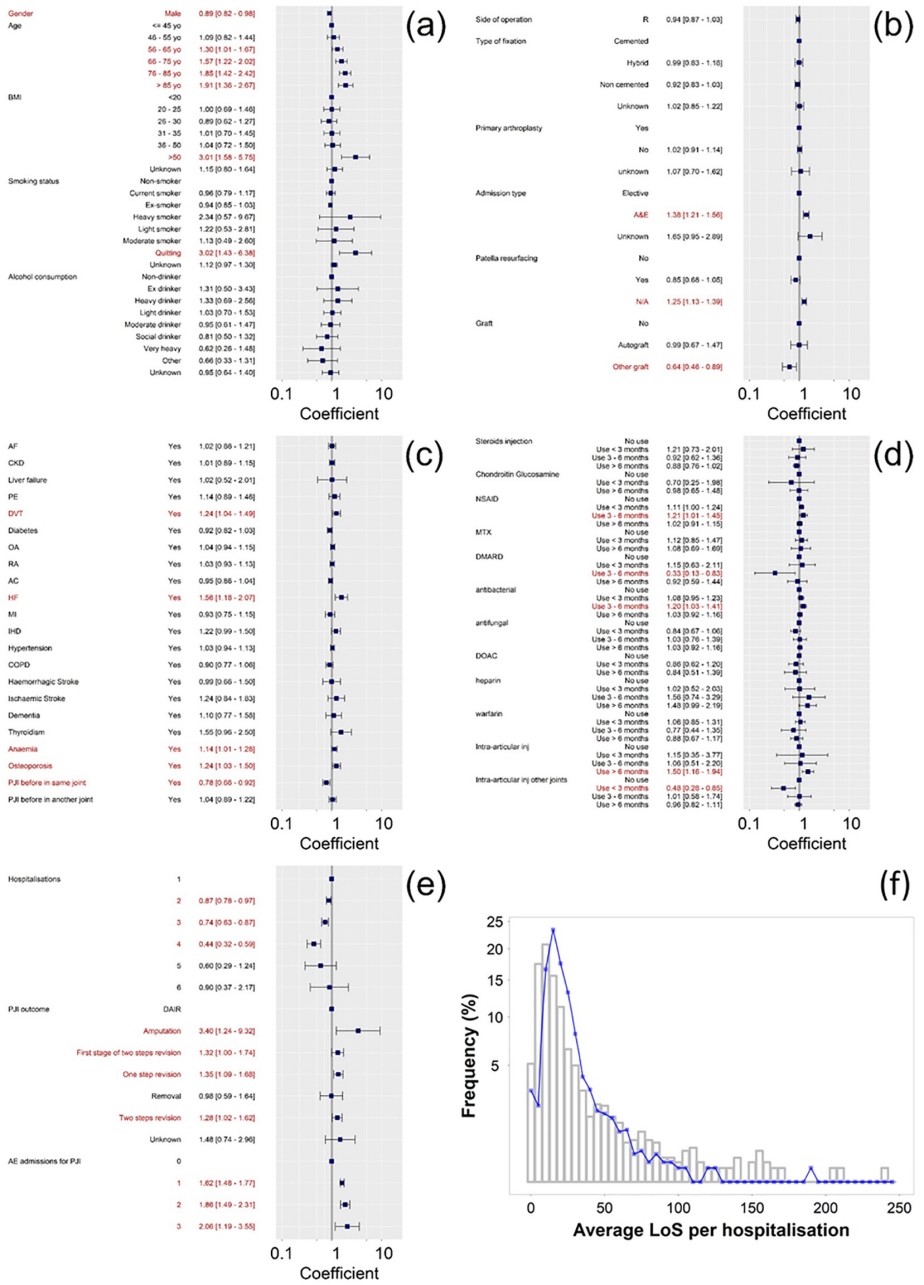
Coefficients for regression of LoS with zero inflated negative binomial distribution for variables related to patient characteristics (a), arthroplasty surgery (b), medical history (c), drug history (d) and PJI characteristics (e). Rootogram of actual LoS and zero inflated negative binomial model predictions (f).

In hospitalizations related to PJI diagnosis, male patients had on average a LoS 10% shorter than women when all other factors were considered; LoS increased with age and was statistically significantly higher for patients with BMI > 50. Average LoS of each hospitalization increased with greater number of admissions through A&E. Patients with PJI also had longer LoS if affected by DVT, HF, anemia or osteoporosis at the time of the joint replacement surgery. When the final outcome of the PJI involved a replacement of the affected device, LoS was longer compared to outcomes with retention of the original device (Fig 3).

The mean number of hospitalizations resulting from the development of PJI was not dependent (p > 0.05) on patients’ characteristics, medical and treatment history or attributes of the initial arthroplasty surgery. LoS regression coefficient regarding PJI outcome were statistically significant (p < 0.05) (S2 Fig).

The diagnosis of PJI resulted in 4,129 procedures associated to its management; the most common recorded procedure was the removal and replacement of the original devices, performed in 1,466 cases; other common procedures performed were aspiration or debridement, amputation was carried out in two cases (Table 5). PJI was diagnosed in a total of 1,654 hospitalizations with 602 following a visit A&E.

**Table 5:** Summary of procedures reported following PJI.

Procedure	Occurrence (n)
Device Removal/replacement	1466
Aspiration	516
Insertion of spacers	516
Debridement/Irrigation	280
Irrigation	233
Superficial debridement	208
Other	199
Drainage	98
Debridement	86
Intra-articular injection	19
Amputation	2

**Fig 4:**
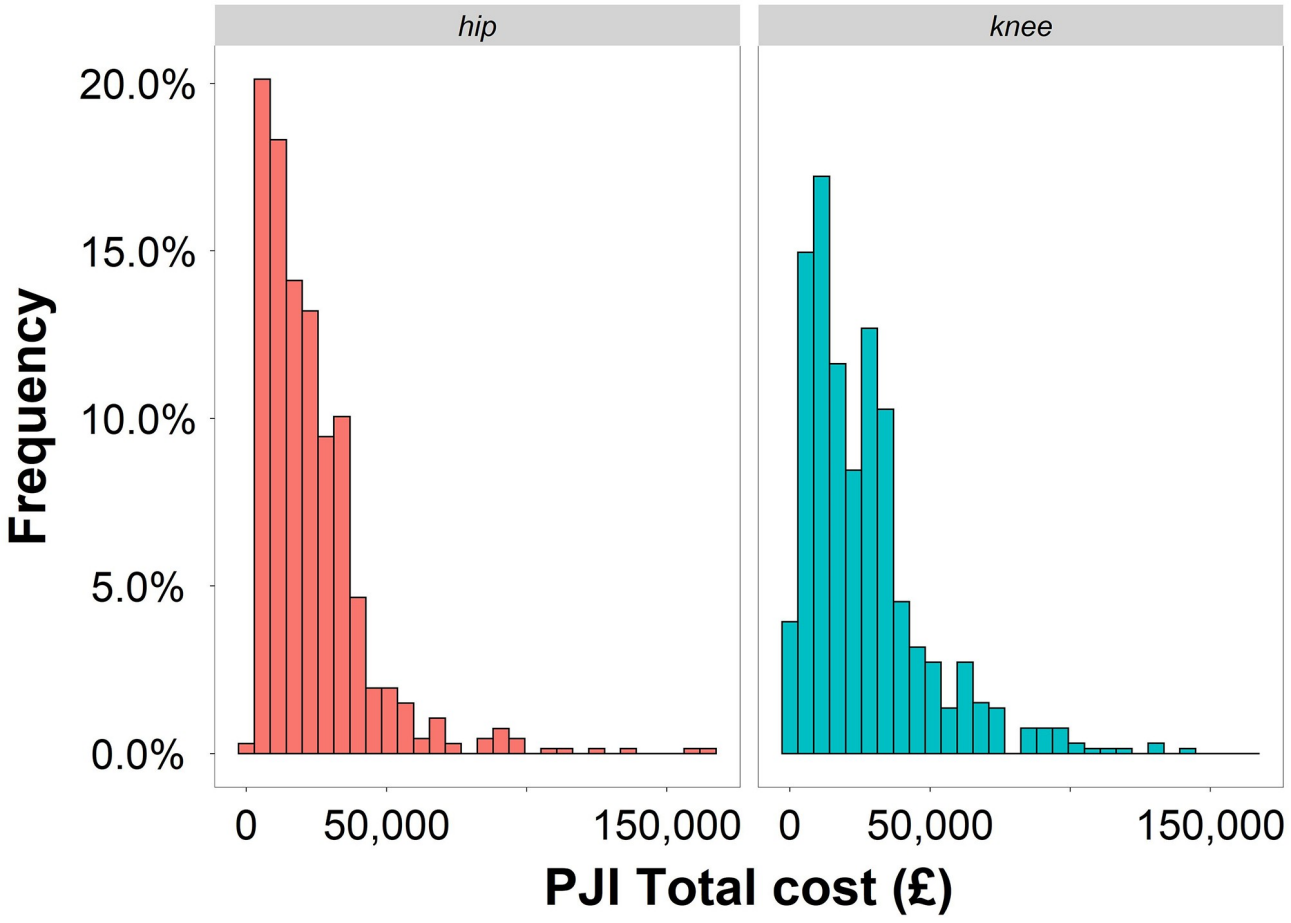
Histograms of direct costs associated with PJIs developed in hips and knees.

The most common final outcome of the recorded PJIs was the revision of the device (performed in one stage for 743 patients and in two stages for 476 patients); the original devise was retained in 95 patients (Table 6). 5.4 and 9.3% of PJI were resolved with DAIR in hip and knee, respectively. The distribution of the direct costs (based on the cost of each procedure performed, type of admission and patients’ comorbidities) associated to each of the recorded PJI revealed a clear not Gaussian profile for both hip and knee devices. The total cost of PJI ranged between £1,146 and £165,824 for hip PJI and between £2,261 and £140,201 for knee PJI; the mean cost of a PJI after hip replacement was £23,337 while after knee £26,523 while the median costs were £17,668 and £20,399 for hip and knee PJI respectively (Fig 4).

**Table 6:** Summary of final outcomes reported following PJI.

Final outcome	Occurrence (n)
Single stage revision	743
Two steps revision	476
DAIR	95
First stage of two steps revision	69
Removal	10
Unknown	7
Amputation	2

PJI in hips resulting in the replacement of the device had a mean cost of £24,078 against £10,361 when managed only through DAIR; similarly, in knee affected by PJI requiring device replacement, the cost was £28,192 against £8,815 when the device was retained.

#### 3.3.2 PJIs causative micro-organisms

**Fig 5:**
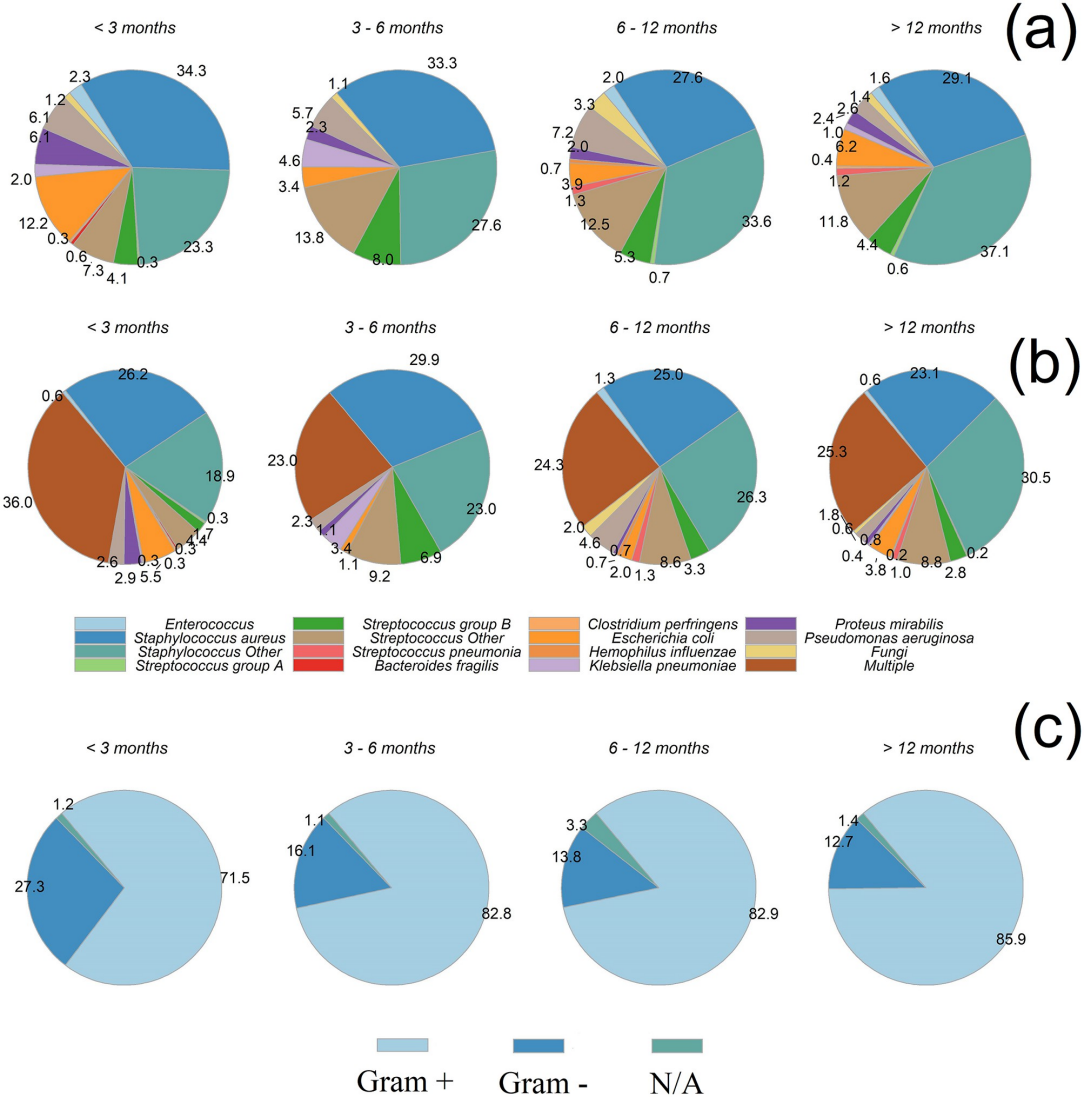
Causative micro-organisms of PJIs observed after different periods of time following arthroplasty aggregated by individual species (a), mono and multi species (b) and Gram (c).

Gram+ bacteria were the common pathogens recorded in PJI at any time after the implant surgery with increasing frequency in late PJI as Gram+ represented 71% of PJI diagnosed in the first 3 months after surgery and over 85% of those diagnosed after more than 12 months from the initial surgery. Regardless of the multi-species or single-species PJI nature, the most common species were *S*. *aureus* and other *Staphylococcus spp*.; *E*. *coli* and *P*. *aeruginosa* were observed in 12.2% and 6.1%, respectively, of PJIs diagnosed in the first 3 months following surgery and in 6.1% and 2.6%, respectively, of PJIs diagnosed more than 12 months following surgery. *Streptococci* represented about 15% of species observed in PJIs at any point after surgery. Multispecies PJIs were observed more frequently in early infections (first 3 months after surgery) than in later PJI (Fig 5).

## 4 Discussion

Most the patients (over 200,000) reached the end of the study period (5 years) and the most common reason for censoring was the termination of the data collection before the end of the study period as expected any patient undergoing arthroplasty after 2015 would not be able to be followed-up for at least 5 years as the data cut for the database extraction was August 2020 The observed cumulative PJI occurrence after 5 years from joint replacement surgery was 0.58% (Fig 1); this is in agreement with the generally reported range of 0.2–1% [34–37]. With the majority of cases developing in the first 6–12 months post device implant and about 2/3 of all cases diagnosed in the first 2 years post-surgery, similarly to other reports [38]. A previous study has employed the England and Wales register along with (HES) database to establish risk factor for PJI [34]. However, this study employed only secondary care information related to patient medical history as HES collects data only in secondary care. This approach limited the covariates considered as information related to patients’ medications and characteristics such as alcohol and smoking habits are collected in primary care in the UK.

### 4.1 PJI risk factors

Male gender and obesity were found risk factors for the development of PJI (Fig 2 and S1 Fig) as also previously reported [34, 38–43]. We observed a decreasing PJI risk with increasing age [34] (Fig 2 and S1 Fig) as also found in other studies [34]; however the impact of age is controversial as an increased risk of PJI for older patients was reported [44] while other studies reported no relation [45, 46].

In terms of medical history, the impact of diabetes on increased rates of PJI was also expected [38, 40–42] as poor glycemic control is generally linked to higher infection rates [47]. When adjusted for other cofactors, the most impactful medical history in PJI risk is a history of previous PJI in the same joint or in another joint, confirming previous findings [48, 49]. Regardless of a previous PJI, a secondary joint replacements was 4 times more likely to develop PJI and in line with previous reported PJI risks after aseptic replacement [50, 51]; this is possibly linked to the higher complexity of the surgery [52]. Moreover, the higher risk of PJI in devices implanted during an unscheduled hospitalization (through A&E) is likely a consequence of the higher complexity of the surgery, tissue damage, greater likelihood of foreign microorganisms entering the patients and often suboptimal pre- and perioperative care in a post-traumatic THA/TKA [53, 54].

The number of intra-articular injections prior to surgery was not included in the multivariable Cox proportional hazard analysis because of the multicollinearity observed with the last injection (no use is equivalent to 0 injections). Despite the modulating/suppressive activity on the immune system, the use of DMARDs was not observed to contribute to PJI development; on the contrary, a prescription of DMARDs in the 3 months prior surgery was associated to a lower risk of PJI (Fig 2); these findings support the recommendation of the International Consensus Meeting on PJI to suspend DMARDs prior to an elective joint arthroplasty based on their half-life [55] and the non-significant role of TNF-α inhibitors prior to knee/hip replacement [43]. We found that steroids use, that also reduce immune system responses, statistically significantly contributed to PJI only when the last prescription was more than 6 months before surgery (Fig 2), partially supporting the concern related to the causality between steroids use and surgical site infections [56]. In contrast with previous finding linking intra-articular injections to higher PJI risk regardless of the time elapsed [57, 58], we did not observe such procedure as a risk factor for PJI. DOACs are normally prescribed for AF or DVT/PE, while such diagnosis was not found to be a risk factor for PJI, only the recent prescription of a DOAC (less than 3 months before arthroplasty) was observed to increase the risk of PJI. Coagulopathy is associated to greater risk of hematoma during surgery and PJI [59]; therefore, a distant prescription is suggestive of a resolved pathology.

The fixation method did not significantly impact the risk of developing PJI in line with previous finding that questioned the long-term efficacy of antibiotic laden bone cement in preventing PJI mainly because of the short-term drug release from such material [60–62].

### 4.2 PJI health resource consumption and outcomes

Zero inflated model returned the best fitting of the LoS associated to a PJI as some patients were more likely to be discharged on the same day they were admitted while zero truncated models performed better in predicting the number of hospitalizations resulting from PJI because no patient with PJI had no hospitalization.

LoS distribution (Fig 3) showed a similar pattern to those of at Nuffield Orthopaedic Centre, Oxford ‘most complex/salvage’ knee with a median LoS = 11 days (IQR 7–19) [5]. Furthermore, we observed longer LoS for hip than knee PJI in agreement with previous [63]. The critical parameters correlated to LoS were gender, age and BMI as generally expected [64–66]. PJI not resulting in device revision had generally shorter LoS because of the less invasive procedures carried out; while patients with history of DVT, anemia and HF spent longer in hospital in line with the general trend of longer LoS in patents with diseases of the circulatory system [67]. Also, attendance to A&E increased LoS likely as in indication of more acute cases.

We observed slightly higher overall mean and median costs for the management of PJI affecting hips than knees. However, the opposite (knee more expensive than hip) was recorded when considering only PJI resulting in the replacement of the original device; this was similar to a previous reports [63, 68]; PJIs that were resolved with DAIR had lower costs because of the reduced complexity of the treatment. The costs observed were similar to those reported in USA [63] but lower compared to other reports of £33,000 [12] and £30,011 [69]. This could be associated to the inclusion of only direct hospital management costs in this study and not the costs related to the diagnosis of PJI and out-patients costs rehabilitation.

Most of the PJI recorded in this study resulted in the replacement of the device or of one the components; however, we observed a higher ratio of single stage revision then previous reports [51]. The observation of final outcomes such as the first stage of a two stages revision were likely the results of two stages replacement in patients lost to follow-up (Table 6).

### 4.3 PJIs causative micro-organisms

Gram+ were the main pathogens observed in PJIs; however, Gram- became more frequent with increasing follow-up time, this is in line with the main difference among early and late PJI. The former infections are predominantly caused by highly virulent pathogens (e.g., *Staphylococcus aureus*, streptococci and enterococci) while the later are mainly caused by low-virulent organisms (e.g., *Propionibacterium acnes*, *S*. *lugdunensis*) [36].

Among the Gram+ pathogens recorded, staphylococci were responsible for more than 50% of PJIs, streptococci and enterococci accounted for about 10% of all PJIs and fungi for about 1–2%; in line with the expected proportions of causative micro-organisms [4, 45, 46, 70, 71].

More than one pathogen species was reported in about a quarter of the recorded PJIs in line with [72] with the greater frequency of polymicrobial species observed in the early PJI as possibly caused by the route of such infection being the surgical wound [4].

### 4.4 Strengths and Limitations of study design, data sources, and analytic methods

This study employed data from the CPRD and HES (admitted patient care/outpatients) databases, as well as death registrations from the ONS allowing for clinical events (for example, diagnoses of PJI, type of management (debridement, revision surgery and amputation) and risk factors (through outpatient/inpatient admissions) to be captured more fully than using either primary care data from the CPRD or secondary care from HES alone. Furthermore, ONS death registrations data ensured accurate and complete recording of all-cause mortality among study patients. A further strength of this study is the large samples of patients reducing uncertainty in the effect size estimated.

This study was retrospective in nature and reliant upon routinely captured data. As with current publications assessing incidence of PJI associated with patient factors, it is likely that a number of plausible confounding variables were unavailable, either because they are not routinely recorded (for example, nutritional status, genetic factors) or unavailable (for example, hospital prescribing and over the counter medication use). The records in the database do not differentiate the presence of antimicrobials in bone cement used during fixation.

Additionally, as we are using register-based data, the possibility of error in the recording of data or codes may be a source of information bias.

As the CPRD is a retrospective observational database, clinical measurements were collected through routine primary care rather than being mandated through a study protocol, as in a prospective study. Missing values, therefore, were present in covariates at baseline.

The study population was restricted to patients with complete data at baseline for certain variables inviting bias, as the patient profile of those with complete data may be different to those without. However, a workable dataset was required in order to fulfil the study objectives and, therefore, this practical approach was adopted to identify a suitable study population.

### 4.5 Recommendation and future directions

The impact of each risk factors on the probability of developing PJI is unlikely to be identical, therefore risk equations capturing the contribution of each factor on the likelihood of PJI would provide further scope for the identification of patients at risk.

Incorporation of time varying covariates would allow a better representation of the patients’ status through the follow-up period particularly for medical history and drug use.

As some potentially relevant risk factors were not available through the chosen data sources, a study containing such information would fill such knowledge gap.

## 5 Conclusions

The knowledge about risks factors for PJI, specific to England and Wales, generated in this study reinforces the role of some commonly accepted drivers while also showed that other sometimes considered influential are not shaping the probability of developing PJI. Thus, this work can provide reasons to update local guidelines.

Additionally, the quantification of the contemporary impact of PJI on resource utilization such as hospital capacity (LoS and A&E admissions) along with the budgetary impact will provide the necessary information for the evaluation of future intervention for the prevention or managements of PJI.

Gram+ were the most common causative microorganisms of PJI; early PJI were more likely to be multispecies than later (years after surgery); the presence of Gram- decreased with increasing post-surgery follow-up.

## Supporting information

S1 Table OPCS codes used to identify arthroplasty surgery. List of OPCS and respective description of codes used for the identification of procedures associated to hip and knee replacement.
(DOCX)

S2 Table OPCS codes used to identify PJI outcomes. List of OPCS and respective description of codes used for the identification of procedures associated to PJI.
(DOCX)

S3 Table Flow of patients through initial data extraction. Number of joints and patients and identified and meeting inclusion criteria.
(DOCX)

S4 Table Number of joints not completing the observation study period (5 years) and reason for loss during follow-up. Number of patients not completing the study for each of the reason considered.
(DOCX)

S5 Table Summary of fitting performance measurement for average LoS for PJI using different distributions. Statistical assessment of fitting LoS with different models.
(DOCX)

S6 Table Summary of fitting performance measurement for number of hospitalisations following PJI using different distributions. Statistical assessment of fitting number of hospitalisatioin with different models.
(DOCX)

S1 Fig Forest plot of Hazard ratios (HR) of univariate Cox proportional regression model for variables related to patient characteristics (a), arthroplasty surgery (b), medical history (c) and drug history (d). (DOCX)

S2 Fig Coefficients for regression of number of hospitalizations following PJI with zero truncated Poisson distribution for variables related to patient characteristics (a), arthroplasty surgery (b), medical history (c), drug history (d) and PJI characteristics (e). Rootogram of actual number of hospitalizations and zero truncated Poisson model predictions (f).
(DOCX)

## Abbreviations

A&E: Accident and Emergency
AC: Active cancer
AF: Atrial Fibrillation
AIC: Akaike Information Criterion
BIC: Bayesian Information Criterion
CKD: Chronic kidney disease
COPD: Chronic obstructive pulmonary disease
DAIR: Debridement, antibiotic implant retention
DMARD: Disease modifying anti-rheumatoid arthritis drug
DOAC: Direct oral anticoagulant
DVT: Deep vein thrombosis
HF: Heart failure
IHD: Ischaemic heart disease
IQR: Interquartile range
LoS: Length of Stay
MI: Myocardial infarction
MTX: Methotrexate
NHS: National healthcare system
NSAID: Non-steroidal anti-inflammatory drug
OA: Osteoarthritis
PE: Pulmonary embolism
RA: Rheumatoid arthritis
TJA: Total joint arthroplasty
