# Supplementary material for: A retrospective study of risk factors, causative micro-organisms and healthcare resources consumption associated with prosthetic joint infections (PJI) using the Clinical Practice Research Datalink (CPRD) Aurum database
Source: PLoS One. 2023 Mar 21;18(3):e0282709. doi: 10.1371/journal.pone.0282709 (PMC10030031; doi:10.1371/journal.pone.0282709)
Supplement: S1 Table — List of OPCS and respective description of codes used for the identification of procedures associated to hip and knee replacement. (DOCX) [file pone.0282709.s001.docx]

Table S 1. OPCS codes used to identify arthroplasty surgery.

| Code | Description |
| --- | --- |
| W37.1 | Primary total prosthetic replacement of hip joint using cement |
| W37.2 | Conversion to total prosthetic replacement of hip joint using cement |
| W37.3 | Revision of total prosthetic replacement of hip joint using cement |
| W37.4 | Revision of one component of total prosthetic replacement of hip joint using cement |
| W37.8 | Other specified |
| W37.9 | Unspecified |
| W38.1 | Primary total prosthetic replacement of hip joint not using cement |
| W38.2 | Conversion to total prosthetic replacement of hip joint not using cement |
| W38.3 | Revision of total prosthetic replacement of hip joint not using cement |
| W38.4 | Revision of one component of total prosthetic replacement of hip joint not using cement |
| W38.8 | Other specified |
| W38.9 | Unspecified |
| W39.1 | Primary total prosthetic replacement of hip joint NEC |
| W39.2 | Conversion to total prosthetic replacement of hip joint NEC |
| W39.3 | Revision of total prosthetic replacement of hip joint NEC |
| W39.5 | Revision of one component of total prosthetic replacement of hip joint NEC |
| W39.8 | Other specified |
| W39.9 | Unspecified |
| W93.1 | Primary hybrid prosthetic replacement of hip joint using cemented acetabular component |
| W93.2 | Conversion to hybrid prosthetic replacement of hip joint using cemented acetabular component |
| W93.3 | Revision of hybrid prosthetic replacement of hip joint using cemented acetabular component |
| W93.8 | Other specified |
| W93.9 | Unspecified |
| W94.1 | Primary hybrid prosthetic replacement of hip joint using cemented femoral component |
| W94.2 | Conversion to hybrid prosthetic replacement of hip joint using cemented femoral component |
| W94.3 | Revision of hybrid prosthetic replacement of hip joint using cemented femoral component |
| W94.8 | Other specified |
| W94.9 | Unspecified |
| W95.1 | Primary hybrid prosthetic replacement of hip joint using cement NEC |
| W95.2 | Conversion to hybrid prosthetic replacement of hip joint using cement NEC |
| W95.3 | Revision of hybrid prosthetic replacement of hip joint using cement NEC |
| W95.8 | Other specified |
| W95.9 | Unspecified |
| W40.1 | Primary total prosthetic replacement of knee joint using cement |
| W40.2 | Conversion to total prosthetic replacement of knee joint using cement |
| W40.3 | Revision of total prosthetic replacement of knee joint using cement |
| W40.4 | Revision of one component of total prosthetic replacement of knee joint using cement |
| W40.8 | Other specified |
| W40.9 | Unspecified |
| W41.1 | Primary total prosthetic replacement of knee joint not using cement |
| W41.2 | Conversion to total prosthetic replacement of knee joint not using cement |
| W41.3 | Revision of total prosthetic replacement of knee joint not using cement |
| W41.4 | Revision of one component of total prosthetic replacement of knee joint not using cement |
| W41.8 | Other specified |
| W41.9 | Unspecified |
| W42.1 | Primary total prosthetic replacement of knee joint NEC |
| W42.2 | Conversion to total prosthetic replacement of knee joint NEC |
| W42.3 | Revision of total prosthetic replacement of knee joint NEC |
| W42.5 | Revision of one component of total prosthetic replacement of knee joint NEC |
| W42.8 | Other specified |
| W42.9 | Unspecified |
| W46.1 | Primary prosthetic replacement of head of femur using cement |
| W46.2 | Conversion to prosthetic replacement of head of femur using cement |
| W46.3 | Revision of prosthetic replacement of head of femur using cement |
| W46.8 | Other specified |
| W46.9 | Unspecified |
| W47.1 | Primary prosthetic replacement of head of femur not using cement |
| W47.2 | Conversion to prosthetic replacement of head of femur not using cement |
| W47.3 | Revision of prosthetic replacement of head of femur not using cement |
| W47.8 | Other specified |
| W47.9 | Unspecified |
| W48.1 | Primary prosthetic replacement of head of femur NEC |
| W48.2 | Conversion to prosthetic replacement of head of femur NEC |
| W48.3 | Revision of prosthetic replacement of head of femur NEC |
| W48.8 | Other specified |
| W48.9 | Unspecified |
| O18.1 | Primary hybrid prosthetic replacement of knee joint using cement NCCS |
| O18.2 | Conversion to hybrid prosthetic replacement of knee joint using cement |
| O18.3 | Revision of hybrid prosthetic replacement of knee joint using cement |
| O18.8 | Other specified |
| O18.9 | Unspecified |
