# Supplementary material for: A retrospective study of risk factors, causative micro-organisms and healthcare resources consumption associated with prosthetic joint infections (PJI) using the Clinical Practice Research Datalink (CPRD) Aurum database
Source: PLoS One. 2023 Mar 21;18(3):e0282709. doi: 10.1371/journal.pone.0282709 (PMC10030031; doi:10.1371/journal.pone.0282709)
Supplement: S2 Table — List of OPCS and respective description of codes used for the identification of procedures associated to PJI. (DOCX) [file pone.0282709.s002.docx]

Table S 2. OPCS codes used to identify PJI outcomes.

| Code | Description | Outcome |
| --- | --- | --- |
| O18.4 | Attention to hybrid prosthetic replacement of knee joint using cement |  |
| W39.4 | Attention to total prosthetic replacement of hip joint NEC |  |
| W42.4 | Attention to total prosthetic replacement of knee joint NEC |  |
| W48.4 | Attention to prosthetic replacement of head of femur NEC |  |
| W80.1 | Open debridement and irrigation of joint | Debridement |
| W80.2 | Open debridement of joint NEC | Debridement |
| W80.3 | Open irrigation of joint NEC | Irrigation |
| W80.8 | Other specified | Debridement |
| W80.9 | Unspecified | Debridement |
| W81.1 | Excision of lesion of joint NEC | Other |
| W81.2 | Open removal of loose body from joint | Other |
| W81.3 | Drainage of joint | Other |
| W81.4 | Incision of joint NEC | Other |
| W81.5 | Exploration of joint NEC | Other |
| W81.6 | Capsulorrhaphy of joint | Other |
| W81.7 | Insertion of therapeutic spacer into joint NCCS | Insertion of spacers |
| W85.2 | Endoscopic irrigation of knee joint | Irrigation |
| W90.1 | Aspiration of joint | Aspiration |
| W90.3 | Injection of therapeutic substance into joint | Intra-articular injection |
| W90.4 | Injection into joint NEC | Intra-articular injection |
| W95.4 | Attention to hybrid prosthetic replacement of hip joint using cement NEC |  |
| X09.1 | Hindquarter amputation | Amputation |
| X09.2 | Disarticulation of hip | Amputation |
| X09.3 | Amputation of leg above knee | Amputation |
| X09.4 | Amputation of leg through knee | Amputation |
| X09.5 | Amputation of leg below knee | Amputation |
| Y03.2 | Renewal of prosthesis in organ NOC | Device Removal/replacement |
| Y03.7 | Removal of prosthesis from organ NOC | Device Removal/replacement |
| Y70.3 | First stage of staged operations NOC | Device Removal/replacement |
| Y70.4 | Primary operations NOC | Device Removal/replacement |
| Y71.1 | Subsequent stage of staged operations NOC | Device Removal/replacement |
| Y71.2 | Secondary operations NOC | Device Removal/replacement |
| S57.1 | Debridement of skin NEC NCCS | Superficial debridement |
| S57.3 | Toilet of skin NEC | Superficial debridement |
| S57.4 | Dressing of skin NEC | Superficial debridement |
| S57.5 | Attention to dressing of skin NEC | Superficial debridement |
| S57.6 | Cleansing and sterilisation of skin NEC | Superficial debridement |
| S57.7 | Dressing of skin using vacuum assisted closure device NEC | Superficial debridement |
| S57.8 | Other specified | Superficial debridement |
| S57.9 | Unspecified | Superficial debridement |
| W69.1 | Synevectomy | Other |
| W69.2 | Synevectomy | Other |
| W69.3 | Synevectomy | Other |
| W69.4 | Synevectomy | Other |
| W69.5 | Synevectomy | Other |
| W37.2 | Conversion to total prosthetic replacement of hip joint using cement | Device Removal/replacement |
| W37.3 | Revision of total prosthetic replacement of hip joint using cement | Device Removal/replacement |
| W37.4 | Revision of one component of total prosthetic replacement of hip joint using cement | Device Removal/replacement |
| W38.2 | Conversion to total prosthetic replacement of hip joint not using cement | Device Removal/replacement |
| W38.3 | Revision of total prosthetic replacement of hip joint not using cement | Device Removal/replacement |
| W38.4 | Revision of one component of total prosthetic replacement of hip joint not using cement | Device Removal/replacement |
| W39.2 | Conversion to total prosthetic replacement of hip joint NEC | Device Removal/replacement |
| W39.3 | Revision of total prosthetic replacement of hip joint NEC | Device Removal/replacement |
| W39.5 | Revision of one component of total prosthetic replacement of hip joint NEC | Device Removal/replacement |
| W93.2 | Conversion to hybrid prosthetic replacement of hip joint using cemented acetabular component | Device Removal/replacement |
| W93.3 | Revision of hybrid prosthetic replacement of hip joint using cemented acetabular component | Device Removal/replacement |
| W94.2 | Conversion to hybrid prosthetic replacement of hip joint using cemented femoral component | Device Removal/replacement |
| W94.3 | Revision of hybrid prosthetic replacement of hip joint using cemented femoral component | Device Removal/replacement |
| W95.2 | Conversion to hybrid prosthetic replacement of hip joint using cement NEC | Device Removal/replacement |
| W95.3 | Revision of hybrid prosthetic replacement of hip joint using cement NEC | Device Removal/replacement |
| W40.2 | Conversion to total prosthetic replacement of knee joint using cement | Device Removal/replacement |
| W40.3 | Revision of total prosthetic replacement of knee joint using cement | Device Removal/replacement |
| W40.4 | Revision of one component of total prosthetic replacement of knee joint using cement | Device Removal/replacement |
| W41.2 | Conversion to total prosthetic replacement of knee joint not using cement | Device Removal/replacement |
| W41.3 | Revision of total prosthetic replacement of knee joint not using cement | Device Removal/replacement |
| W41.4 | Revision of one component of total prosthetic replacement of knee joint not using cement | Device Removal/replacement |
| W42.2 | Conversion to total prosthetic replacement of knee joint NEC | Device Removal/replacement |
| W42.3 | Revision of total prosthetic replacement of knee joint NEC | Device Removal/replacement |
| W42.5 | Revision of one component of total prosthetic replacement of knee joint NEC | Device Removal/replacement |
| W46.2 | Conversion to prosthetic replacement of head of femur using cement | Device Removal/replacement |
| W46.3 | Revision of prosthetic replacement of head of femur using cement | Device Removal/replacement |
| W47.2 | Conversion to prosthetic replacement of head of femur not using cement | Device Removal/replacement |
| W47.3 | Revision of prosthetic replacement of head of femur not using cement | Device Removal/replacement |
| W48.2 | Conversion to prosthetic replacement of head of femur NEC | Device Removal/replacement |
| W48.3 | Revision of prosthetic replacement of head of femur NEC | Device Removal/replacement |
| O18.2 | Conversion to hybrid prosthetic replacement of knee joint using cement | Device Removal/replacement |
| O18.3 | Revision of hybrid prosthetic replacement of knee joint using cement | Device Removal/replacement |
| Y22.1 | Aspiration of haematoma of organ NOC | Drainage |
| Y22.2 | Aspiration of other lesion of organ NOC | Drainage |
| Y22.3 | Irrigation of organ NOC | Drainage |
