# Supplementary material for: A retrospective study of risk factors, causative micro-organisms and healthcare resources consumption associated with prosthetic joint infections (PJI) using the Clinical Practice Research Datalink (CPRD) Aurum database
Source: PLoS One. 2023 Mar 21;18(3):e0282709. doi: 10.1371/journal.pone.0282709 (PMC10030031; doi:10.1371/journal.pone.0282709)
Supplement: S3 Table — Number of joints and patients and identified and meeting inclusion criteria. (DOCX) [file pone.0282709.s003.docx]

Table S 3. Flow of patients through initial data extraction.

|  | Number of joints | Number of patients |
| --- | --- | --- |
| Knee or hip replacement in database | 330,173 | 235,249 |
| Over 31 years old at index date | 329,720 | 235,071 |
| Arthroplasty surgery after 2007 | 288,124 | 223,450 |
| With minimum 6 months lookback | 284,048 | 222,060 |
| After removing implausible data | 283,789 | 221,826 |
