# Supplementary material for: A retrospective study of risk factors, causative micro-organisms and healthcare resources consumption associated with prosthetic joint infections (PJI) using the Clinical Practice Research Datalink (CPRD) Aurum database
Source: PLoS One. 2023 Mar 21;18(3):e0282709. doi: 10.1371/journal.pone.0282709 (PMC10030031; doi:10.1371/journal.pone.0282709)
Supplement: S4 Table — Number of patients not completing the study for each of the reason considered. (DOCX) [file pone.0282709.s004.docx]

Table S 4. Number of joints not compleating the observation study period (5 years) and reason for loss during follow-up.

| Reason for not completing study period | n | Frequency |
| --- | --- | --- |
| Developing PJI | 1,402 | 0.49% |
| Death | 32,981 | 11.62% |
| Last collection date before end of observation period | 76,800 | 27.06% |
| Device replaced for any reason | 5,371 | 1.89% |
| Transferred out of CPRD | 35,831 | 12.63% |
