# Supplementary material for: A retrospective study of risk factors, causative micro-organisms and healthcare resources consumption associated with prosthetic joint infections (PJI) using the Clinical Practice Research Datalink (CPRD) Aurum database
Source: PLoS One. 2023 Mar 21;18(3):e0282709. doi: 10.1371/journal.pone.0282709 (PMC10030031; doi:10.1371/journal.pone.0282709)
Supplement: S5 Table — Statistical assessment of fitting LoS with different models. (DOCX) [file pone.0282709.s005.docx]

Table S 5. Summary of fitting performance measuremement for average LoS for PJI using different distributions.

| Model | Log-likelihood | AIC | BIC |
| --- | --- | --- | --- |
| Negative binomial | -11,669.4 | 23,568.8 | 24,172.0 |
| Zero inflated negative binomial | -5,422.1 | 11,076.2 | 11,684.6 |
| Poisson | -11,166.8 | 22,787.6 | 23,978.2 |
| Zero inflated Poisson | -5,258.7 | 10,973.4 | 12,169.3 |
