# Supplementary material for: A retrospective study of risk factors, causative micro-organisms and healthcare resources consumption associated with prosthetic joint infections (PJI) using the Clinical Practice Research Datalink (CPRD) Aurum database
Source: PLoS One. 2023 Mar 21;18(3):e0282709. doi: 10.1371/journal.pone.0282709 (PMC10030031; doi:10.1371/journal.pone.0282709)
Supplement: S6 Table — Statistical assessment of fitting number of hospitalisatioin with different models. (DOCX) [file pone.0282709.s006.docx]

Table S 6. Summary of fitting performance measuremement for number of hospitalisations following PJI using different distributions.

| Model | Log-likelihood | AIC | BIC |
| --- | --- | --- | --- |
| Poisson | -1,763.6 | 3,745.2 | 4,316.9 |
| Zero truncated Poisson | -1,169.8 | 2,557.5 | 3,129.2 |
