# Supplementary material for: A retrospective study of risk factors, causative micro-organisms and healthcare resources consumption associated with prosthetic joint infections (PJI) using the Clinical Practice Research Datalink (CPRD) Aurum database
Source: PLoS One. 2023 Mar 21;18(3):e0282709. doi: 10.1371/journal.pone.0282709 (PMC10030031; doi:10.1371/journal.pone.0282709)
Supplement: S1 Fig — (DOCX) [file pone.0282709.s007.docx]

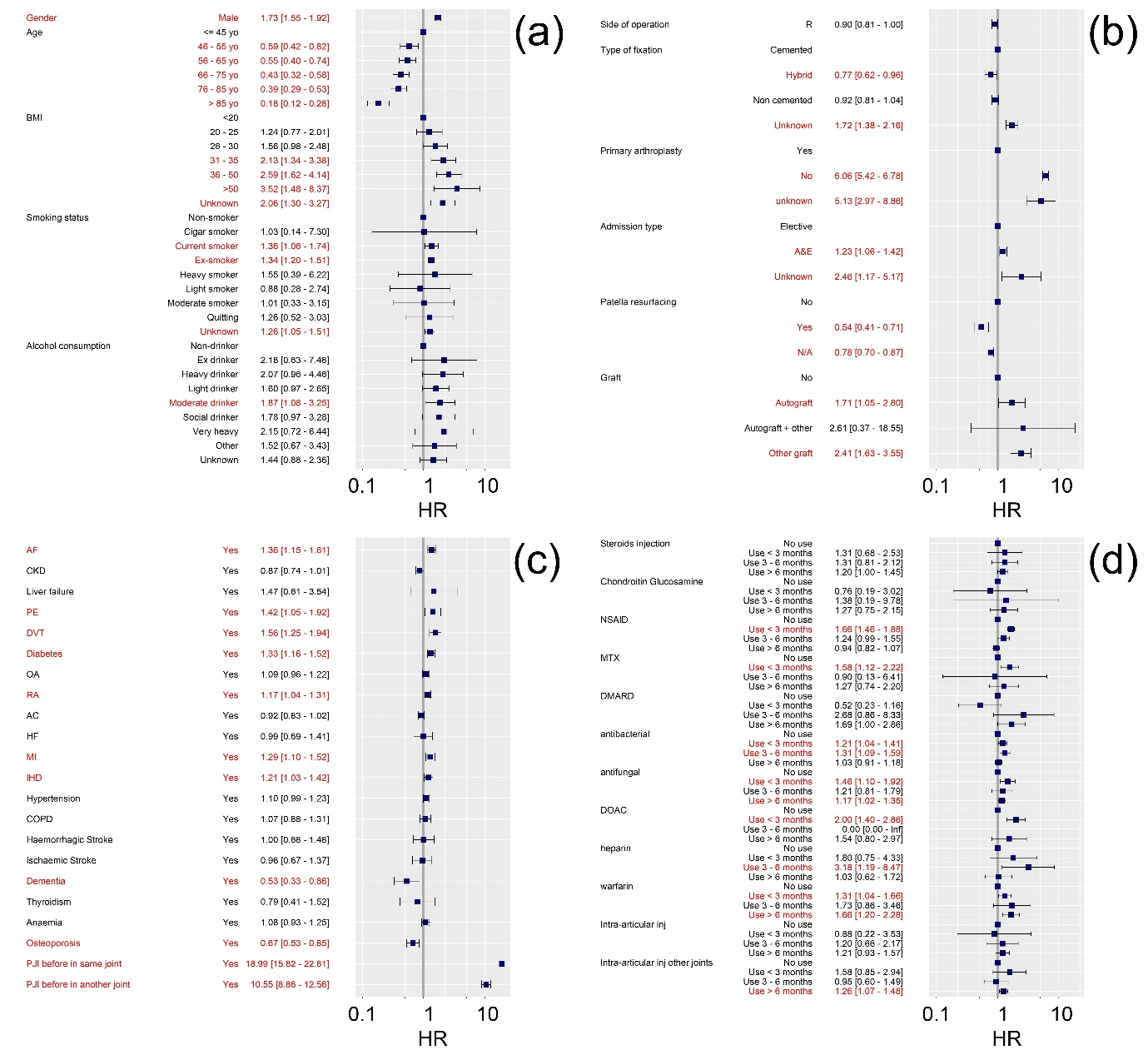


Figure S 1. Forest plot of Hazard ratios (HR) of univariate Cox proportional regression model for variables related to patient characteristics (a), arthroplasty surgery (b), medical history (c) and drug history (d).
