# Supplementary material for: A retrospective study of risk factors, causative micro-organisms and healthcare resources consumption associated with prosthetic joint infections (PJI) using the Clinical Practice Research Datalink (CPRD) Aurum database
Source: PLoS One. 2023 Mar 21;18(3):e0282709. doi: 10.1371/journal.pone.0282709 (PMC10030031; doi:10.1371/journal.pone.0282709)
Supplement: S2 Fig — Coefficients for regression of number of hospitalizations following PJI with zero truncated Poisson distribution for variables related to patient characteristics (a), arthroplasty surgery (b), medical history (c), drug history (d) and PJI characteristics (e). Rootogram of actual number of hospitalizations and zero truncated Poisson model predictions (f). (DOCX) [file pone.0282709.s008.docx]

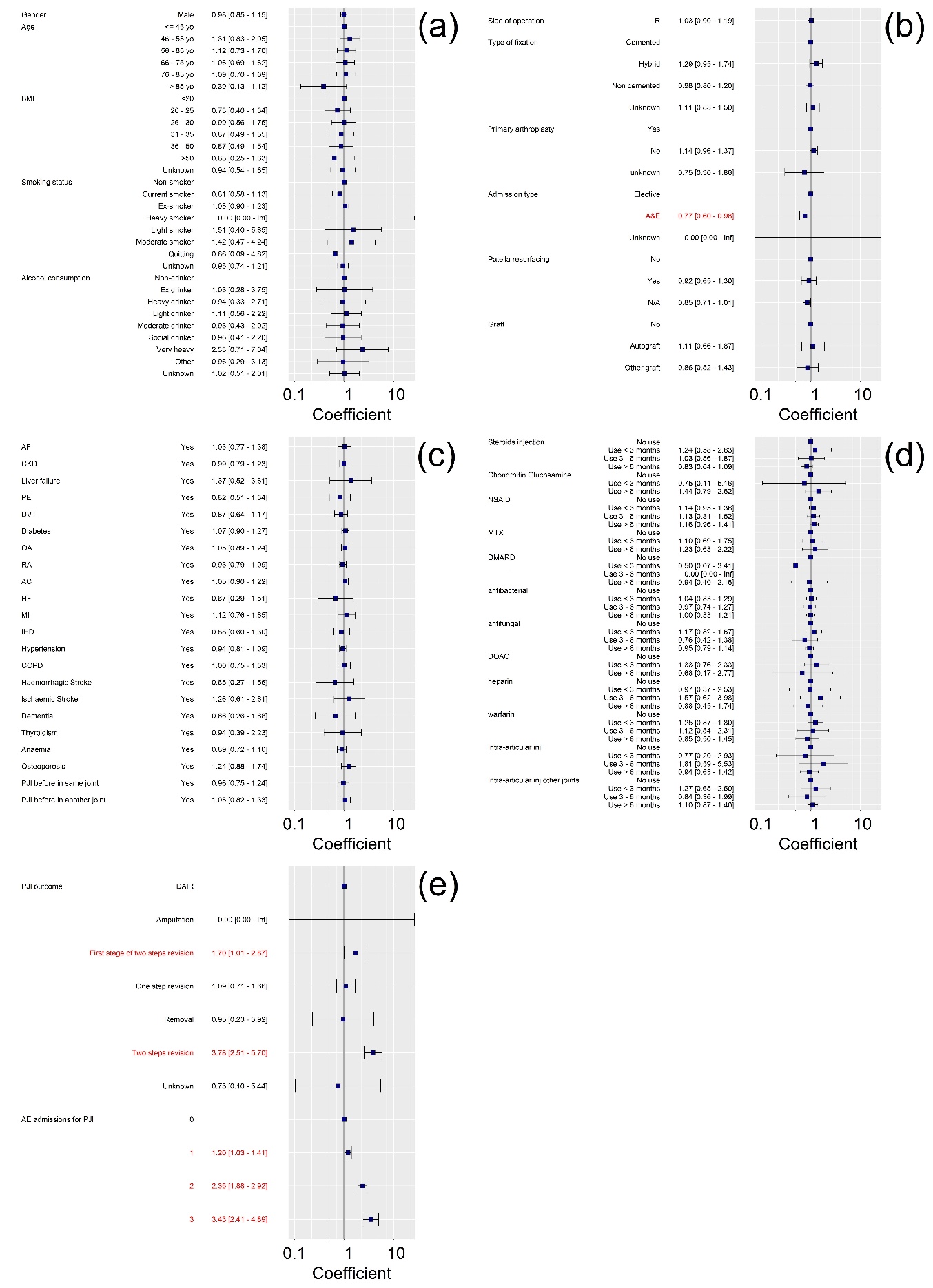


Figure S 2. Coefficients for regression of number of hospitalizations following PJI with zero truncated Poisson distribution for variables related to patient characteristics (a), arthroplasty surgery (b), medical history (c), drug history (d) and PJI characteristics (e). Rootogram of actual number of hospitalizations and zero truncated Poisson model predictions (f).
